# Transformation of primary care settings implementing a co-located team-based care model: a scoping review

**DOI:** 10.1186/s12913-024-11291-7

**Published:** 2024-08-05

**Authors:** Yasmine Frikha, Andrew R. Freeman, Nancy Côté, Claudèle Charette, Maxime Desfossés

**Affiliations:** 1 Faculty of Graduate and Post-Doctoral Studies, Université Laval , Québec, Canada; 2 VITAM: Centre de Recherche en Santé Durable, Québec, Québec Canada; 3 School of Rehabilitation Sciences (Faculty of Medicine), Université Laval, Québec, Québec Canada; 4 Department of Sociology (Faculty of Social Sciences), Université Laval, Québec, Québec Canada

**Keywords:** Primary care, Team-based care model, Interprofessional, Scoping review, Family physician, Organization, Role optimization

## Abstract

**Background:**

In Canada, primary care reforms led to the implementation of various team-based care models to improve access and provide more comprehensive care for patients. Despite these advances, ongoing challenges remain. The aim of this scoping review is to explore current understanding of the functioning of these care models as well as the contexts in which they have emerged and their impact on the population, providers and healthcare costs.

**Methods:**

The Medline and CINAHL databases were consulted. To be included, team-based care models had to be co-located, involve a family physician, specify the other professionals included, and provide information about their organization, their relevance and their impact within a primary care context. Models based on inter-professional intervention programs were excluded. The organization and coordination of services, the emerging contexts and the impact on the population, providers and healthcare costs were analysed.

**Results:**

A total of 5952 studies were screened after removing duplicates; 15 articles were selected for final analysis. There was considerable variation in the information available as well as the terms used to describe the models. They are operationalized in various ways, generally consistent with the *Patient’s Medical Home* vision. Except for nurses, the inclusion of other types of professionals is variable and tends to be associated with the specific nature of the services offered. The models primarily focus on individuals with mental health conditions and chronic diseases. They appear to generally satisfy the expectations of the overarching framework of a high-performing team-based primary care model at patient and provider levels. However, economic factors are seldom integrated in their evaluations.

**Conclusions:**

The studies rarely provide an overarching view that permits an understanding of the specific contexts, service organization, their impacts, and the broader context of implementation, making it difficult to establish universal guidelines for the operationalization of effective models. Negotiating the inherent complexity associated with implementing models requires a collaborative approach between various stakeholders, including patients, to tailor the models to the specific needs and characteristics of populations in given areas, and reflection about the professionals to be included in delivering these services.

## Background

Primary care is defined by the World Health Organization (WHO) as a “model of care that supports first-contact, accessible, continuous, comprehensive, and coordinated person-focused care” [1]. A model of care is a conceptualization and operationalization of how services are delivered, encompassing care processes, organization of providers, management of services and enabling factors to support their development [2]. Primary care serves as an accessible entry point for healthcare, providing ongoing care for patients. Primary care also emphasizes prevention and intersectoral collaboration, allowing consideration of the social determinants of health to provide comprehensive treatment [3].

In Canada, primary care services are mostly provided by family physicians and nurse practitioners in family physician clinics, walk-in clinics or privately funded services (e.g., insurer-paid) [4]. Primary care providers detect and treat common health issues, monitor chronic diseases, and as necessary, refer patients to medical specialists and other healthcare services [4]. In the early 2000s, reforms in primary care were implemented to enhance access to quality healthcare. According to the *Quintuple Aim*, the indicators of quality healthcare include improving patient experience, population health and healthcare provider satisfaction, reducing healthcare costs, and ensuring health equity [3, 4]. The Canadian reforms aimed to provide integrated care, facilitate access to psychosocial services, prioritize prevention programs, and address the management of chronic diseases [3, 5, 6]. In 2004, the federal ministry of health set a goal that by 2011, 50% of Canadians should have 24/7 access to a multidisciplinary primary health care team [7]. This objective led to the emergence of various primary care groups and networks, for example, the *Groupes de médecine familiale* (Family Medicine Groups, FMG) in the province of Quebec and the Family Health Teams (FHT) in the province of Ontario [3]. These models are considered co-located team-based primary care models; that is, an interprofessional team collaborates with family physicians in the same location, regardless of the services provided (e.g., mental health care, medical care). These models require the optimization of professional roles so that the right care is delivered by the appropriate professional according to their expertise [8, 9]. Optimizing professional roles means that professionals mobilize their expertise to a greater extent consistent with their respective scope of practice [9, 10]. Role optimization relies on strong interprofessional collaboration within the team to provide effective and comprehensive care [11]. Models of care resulting from the primary care reform, such as the FMG or FHT, were anchored in the *Patient’s Medical Home* (PMH) vision, which focuses on delivering quality care to patients throughout their lives [12]. In this vision, the family physician plays a central role as the principal primary care provider. To ensure the successful functioning of the model consistent with this vision and the accessibility of care, an interprofessional team working in the same physical space with optimized professional roles collaborates with family physicians to support them in providing prevention and health promotion, psychosocial services and chronic disease management to the general population. The patient can consult an appropriate professional, even if it's not their family physician, as collaboration and communication within the team keep the family physician involved as needed, ensuring effective care coordination. Other models based on interprofessional collaboration exist. However, these models tend to be coordinated intervention programs designed to deal with specific problems in certain primary care settings, for example, a program for people with chronic obstructive pulmonary disease [13] or an intervention for depression, diabetes or cardiovascular diseases [14]. These interprofessional interventions differ from the regular services offered in FMGs, possibly cutting across several primary care settings, albeit still requiring collaboration with family physicians.

Although FMGs and FHTs represent examples of significant advancements in the evolution of Canadian primary care services, including their alignment with the PMH perspective, further progress is required, as the initial objectives of these models have not been entirely achieved. For example, with respect to FMGs, a considerable proportion of the population in Quebec still lacks access to a family physician, hospital emergency departments are not adequately relieved of congestion, and access to psychosocial services remains limited [15]. Given these ongoing challenges, it is important to better understand innovative co-located primary care team-based models to identify what is effective, with a view to identifying elements that will contribute towards better meeting the needs of the populations in Quebec, in other Canadian jurisdictions, and elsewhere.

Some reviews have been published that deepen the understanding of primary care models that involve different forms of co-located team-based care [16–19]; some promising results have emerged. For example, Norful and colleagues [19] demonstrated that there is a significantly better adherence to recommended care guidelines within models where there is co-management between nurse practitioners (NPs) and physicians. A reduction in health services utilization (e.g., emergency room visits) has been demonstrated in some investigations when patients are managed within team-based care models [17, 18]. Despite the usefulness of these findings, these reviews generally focus on a single dimension of the models. When the focus is on an organisational model for a given population, the results tend to concern population health outcomes [16, 18], and more rarely economic outcomes [17], and do not describe how each model works, which makes it difficult to reproduce them. Conversely, when the research focuses more specifically on how the model functions with respect to interprofessional collaboration [20] or on the context in which the models are implemented [21], the evaluation of the impact of these models on the population and healthcare is rarely included, which makes it difficult to assess their performance. Longhini and colleagues’ [22] review adopted a more integrated approach by presenting elements of the functioning of the different models of care identified and the impact on the population and healthcare. However, this review’s findings are limited to people with chronic illnesses, and do not provide information on the contexts in which the models are implemented.

Thus, the existing reviews have rarely provided an integrated overview of the composition of teams, the organization of the model of care, details of the context in which the model of care was implemented, and the impact on the population, professionals and the economy of the healthcare system. To facilitate the development and improvement of team-based care models, it is essential to have a better understanding of the functioning of these models and to have an overall vision that considers the inherent complexity of how these models work. Consistent with our earlier definition of model of care [2], functioning refers to the selection and organization of services (e.g., care processes, type of providers), the management of services, and enabling factors (e.g., financing, resources, system to support collaboration and improve quality).

In this article we report the findings of a scoping review of the studies that have examined the functioning of co-located team-based primary care models in their specific contexts. This review includes three sub-objectives:

1. Identify how these co-located models are operationalized within environments with respect to service organization and coordination;
2. Identify in response to which needs and/or in which contexts these models have emerged;
3. Identify the impact of these models on the population, providers and healthcare costs.

## Methods

We chose to carry out a scoping review because this approach permits the development of a portrait of the literature on a relatively vast subject [23], which is relevant given our goal of obtaining an overview of existing co-located team based primary care models, without restricting ourselves to a specific population or a particular context. We adhered to Arksey and O’Malley’s [23] guidelines in conjunction with Levac and colleagues’ [24] approach to guide this scoping review, which follows five steps: (1) identify the research question; (2) identify relevant studies; (3) select study by specifying inclusion and exclusion criteria; (4) charting the data; (5) collating, summarizing and reporting the results.

### Identifying the research question

The research question that guided this scoping review is as follows: How do co-located primary care models based on interprofessional collaboration function in their specific context?

### Identifying relevant studies and study selection

Our initial search was conducted in May 2022, and was subsequently updated in October 2023. Consistent with the socio-medical nature of our research question, we searched the MEDLINE and CINAHL databases, which cover health and other topics related to health care disciplines. We used the following three main keywords, including their variations and the appropriate thesaurus for each database, in English, to identify relevant studies: 'primary care,' 'interprofessional collaboration’, 'model '.

We focused on the concept of interprofessional collaboration, as this concept is broader than role optimization, with the latter relying on interprofessional collaborative practices. The addition of the concept *model* was made with a focus on models of care and to exclude intervention programs as well as the sole description of interprofessional collaborative practices. This search strategy was reviewed by a librarian, who informed us about the variability of terms and their meanings in the literature, thus increasing the precision of our search strategy and better ensuring that we captured all potentially relevant articles. The detailed search strategy used for MEDLINE (conducted in EBSCOhost) is available in Table 1.

**Table 1:** Research strategy used in EBSCOhost for MEDLINE database

1	TI ("Primary care" OR "Primary health care" OR Frontline OR "general practi*" OR "family practi*" OR community) OR AB ("Primary care" OR "Primary health care" OR Frontline OR "general practi*" OR "family practi*" OR community)
2	(MH"Primary Health Care") OR (MH"Primary care Nursing") OR (MH"physicians, primary Care")
3	1 OR 2
4	TI (Collaborat* OR Interprofessional OR "team-based" OR optimis* OR optimiz* OR "integrated care" OR cooperati*) OR AB (Collaborat* OR Interprofessional OR "team-based" OR optimis* OR optimiz* OR "integrated care" OR cooperati*)
5	(MH"Intersectoral Collaboration") OR (MH"Interprofessional relations") OR (MH"Cooperative Behavior")
6	4 OR 5
7	TI ((Model* N2 ("evidence based" OR fund* OR care OR clinic OR organi?ational)) OR AB ((Model* N2 ("evidence based" OR fund OR care OR clinic OR organi?ational))
8	(MH"Models, Organizational")
9	7 OR 8
FINAL	3 AND 6 AND 9

Two reviewers conducted the initial screening (reviewing titles and abstracts) based on inclusion and exclusion criteria presented in the next section. Subsequently, the selected articles were read integrally. To ensure rigour in this process, the two reviewers independently performed both screenings and subsequently consulted with each other to reach a consensus about the articles to be included. In case of any impasse, a third reviewer was involved. Because of the variability of the concepts’ definitions used in the articles, the three reviewers met regularly to ensure a common understanding of each article. During the screening, it was necessary to refine the inclusion and exclusion criteria due to several nuances encountered in our readings. Two additional researchers were consulted as needed to verify the analysis of the selected articles given the complexity of the subject.

### Selecting inclusion and exclusion criteria

The following inclusion and exclusion criteria were applied: (1) Empirical research that evaluated a primary care team-based model; we excluded research protocols and literature reviews. (2) The co-located team-based model had to be operationalized in a primary care context; any articles focused on, for example, hospital settings were excluded. (3) Given our interest in models of care involving the integration of healthcare professionals co-located with family physicians to support primary care provision, models based on inter-professional intervention programs across several primary care settings were excluded. (4) An interprofessional team utilizing the co-located collaboration model had to be in operation within the care setting. (5) Because our interest lies in models similar to FMGs and FHTs that rely on the collaboration and the optimization of roles between a family physician and other professionals, the team had to include a family physician, and the articles had to clarify the types of professionals present. (6) The studies had to include assessment of the impact of the models on the population, professionals and/or costs for the healthcare system. In this regard, articles that solely described collaboration dynamics, interprofessional collaboration skills, and the introduction of a new professional, were not included. (7) Consistent with our focus on the Canadian context, we only included studies conducted in countries with a predominantly Western culture (e.g., Western Europe, North America, Australia, New Zealand) given their greater potential applicability to this context. (8) Studies were published since 2000, based on the relatively recent evolution in primary care models. (9) Studies were published in English or French, given the authors’ mastery of both languages.

### Charting the data

We conducted an initial data extraction by charting the selected articles to identify key elements relevant to our research objectives. First, we highlighted the practice setting to understand the type of primary care organization (e.g., family medicine clinic, community center). Subsequently, to understand how the models are operationalized with respect to the organization and coordination of services (Objective 1), keeping in mind our interest in optimizing professional roles, we extracted information relating to: the type of professionals present in each of the teams; the principle orientation of the services offered (e.g., mental health, chronic illness); the access methods to these professionals and the patient's trajectory in the setting; the systems used to support collaboration and communication (e.g., use of a shared electronic medical record, patient registry, interprofessional meetings) and the type of management. This data will help to document the selection, organization and management of services, especially regarding interprofessional team-based organization [2]. Communication, which is supported by the use of technology, is an essential part of team-based organization [25]. We also identified the model of care approach, as described by the authors; this approach refers to the overall vision that underpins the design and delivery of healthcare services based on patient needs and clinical best practices [26]. Subsequently, we created a category to collect data regarding the contexts in which these models emerged (Objective 2). Specifically, we extracted data related to the socio-professional context, the socio-demographic characteristics of the population in the target area, and the nature and origin of the support received for the model's operation (e.g., provincial funding, a local initiative supported by the city). Understanding local contexts and identifying the specific enabling factors within them permits a clearer comprehension of how these models were adapted to meet local needs, opportunities, and constraints [27]. We also identified the methodology used in each selected article, including objectives, data collection methods, and sample characteristics. Last, we extracted the main results of the studies documenting the impact of the models on the population, professionals and costs for the healthcare system (Objective 3).

### Collating, summarizing and reporting the results

Two reviewers independently created summaries of the gathered information and subsequently compared their respective versions to reach a consensus, ensuring the quality of the retained data. In case of any conflicts, a third person helped resolve the impasse. This process made it possible to synthesize the considerable volume of information extracted and standardize the presentation of the data so that it could be compared. We reported the results according to our three sub-objectives, that is: (1) information that provides a clearer understanding of the organization and coordination of services; (2) information relating to the contexts with the aim of understanding the characteristics of the sector in which the models were implemented and the factors that led to their development; (3) the results relating to the impact of the model and the methodological aspects of the research, in order to make sense of these results.

## Results

### Description

**Fig. 1:**
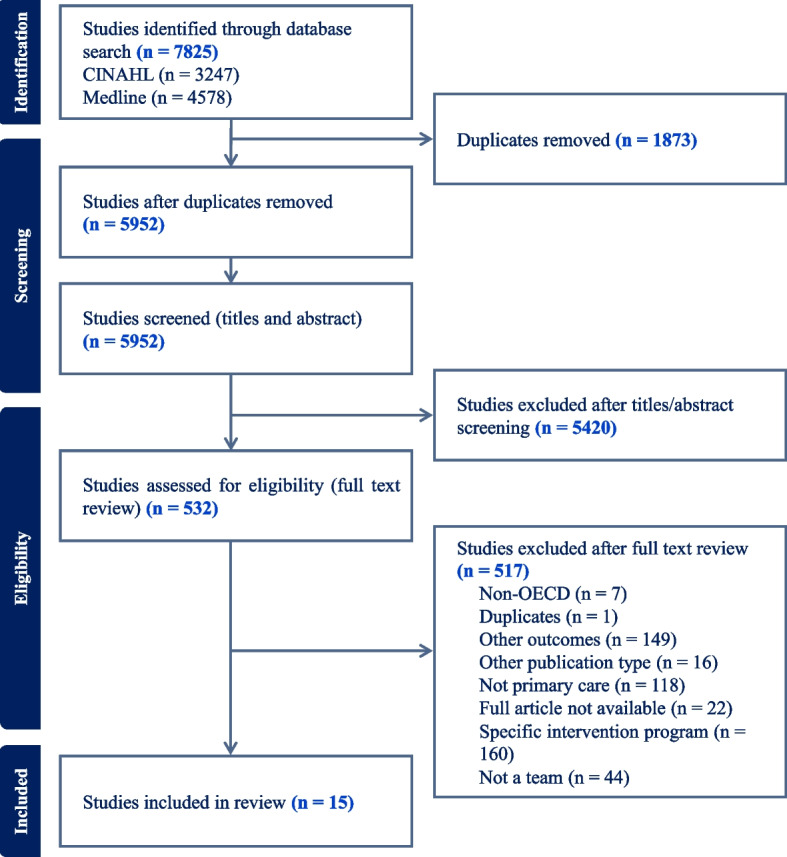
Prisma flow

A total of 5952 studies were screened following the removal of duplicates. Subsequently, 5420 studies were excluded following title and abstract screening, leaving 532 articles requiring full text screening. Following the exclusion of 517 documents, 15 were included in the scoping review. The detailed selection process, including the reasons for exclusion, is shown in Fig. 1. The significant reduction is explained by the fact that most team-based models are coordinated intervention programs designed to treat specific problems (e.g., diabetes, Chronic Obstructive Pulmonary Disease, depressive symptoms, dementia) rather than models of care based on the provision by co-located interprofessional teams of services for a range of conditions (see “Limitations of the study” for further details). Moreover, there was considerable variability regarding the definition of the concept of team, team-based care model, interprofessional teams or integrated care. The team-based models reported have been implemented mostly in the United-States and Canada (13/15; 86,7%).

### Model of care approach

Nearly all the studies referred to the model of care approach upon which the practice was implemented in their settings. In Table 2, these approaches are presented using the study authors’ definitions provided in their article. When the underlying approach was developed by other authors, the reference is indicated in the first column. In this case, the original definition was provided. Although not consistently stated explicitly by the authors, in all team-based models, complementary services and primary care are delivered in the same location [8]. Across the studies, there was considerable variability in the approaches used and how they were defined. This variability reflects different ways of organizing services. For example, multiple variations of a collaborative care model have been presented. Among these variations, one approach highlights the integration of a care manager [28], another the development of the nurse practitioner role [29], while another emphasizes the nature of interprofessional collaboration [30]. The most cited approach is the Canadian shared-care model (3/15; 20%) designed by Kates and colleagues [31].

**Table 2:** Models of care approaches

Approach	Description provided	Country, Articles
Basic collaboration on site (co-located), [60]	Mental health and other health care professionals have separate systems but share the same facility. They engage in regular communication about shared patients, mostly through telephone or letters, but occasionally meet face to face because of their proximity [60]	USA Moore et al. (2018) [ 35 ]
Chronic care model [61]	An approach that combines delivery system redesign, enhanced use of technology for real-time decision making, and patient self-management support to produce more productive interactions and thus better outcomes [61]	USA Lyon & Slawson (2011) [ 37 ]; Scanlon et al., 2008 [ 39 ]
Close collaboration in a fully integrated system (integrated care) [60]	Mental health and other health care professionals share the same sites, the same vision, and the same systems in a seamless web of biopsychosocial services. Regular collaborative team meetings are held to discuss both patient issues and team collaboration issues [60]	USA Moore et al. (2018) [ 35 ]
Collaborative care	A process whereby primary care and mental health providers share resources, expertise, knowledge and decision-making to ensure that primary care populations receive person-centred, effective, and cost-effective care from the right provider in the most convenient location and in the most timely and well-coordinated manner. [30]	Norway Rugkåsa et al. (2020) [ 30 ]
Collaborative care model (a)	Team-based approach that can more efficiently meet population health care demands and individual patient needs through collaborative management. Behavioral health teams, including a care manager, monitor patient progress and outcomes through measurement-informed care facilitated by a patient registry and provide timely treatment adjustment and regular psychiatric case reviews to primary care providers [28]	USA Blackmore et al. (2018) [ 28 ]
Collaborative care model (b)	Based on the development of the role and implementation of a nurse practitioner in the practice, a new physical infrastructure, the development of a mission statement, and formalized evaluation activities [29]	Canada Lawson et al. (2012) [ 29 ]
Co-located	A behavioral health specialist works in the same practice as the primary care team. Patients may be identified through screening or clinical recognition of symptoms, and the primary care provider typically refers patients to one or more behavioral health providers for evaluation and treatment [28]	USA Blackmore et al. (2018) [ 28 ]
Comprehensive integrated clinic	Care is provided in a shared practice space and collaboration is driven by team-based care [40]	USA Emerson et al., (2023) [ 40 ]
Family Health Teams [12]	Family health teams are Ontario’s model of transformed primary care derived from the PMH [12]	Canada Ashcroft et al. (2021) [ 32 ]
Integrated behavioral health	A collaborative care model of identifying and treating mild to moderate mental disorders in adults in a primary care setting [36]	USA Sanchez & Adorno (2013) [ 36 ]
Integrated care	The essential feature of integrated care is its commitment to overcoming fragmented care, and to meeting complex care needs through ongoing and co-productive partnerships [33]	Australia Fitzpatrick et al. (2018) [ 33 ]
Patient’s Medical Home, [12]	Team-based model of primary care where family physicians work in tandem with interprofessional teams to provide continuous and coordinated person-centred care [12]	Canada Ashcroft et al. (2021) [ 32 ]
Shared care model [31]	A close collaboration between family physicians and mental health professionals in primary care is enhanced, providing support to family physicians in the care of their patients [31]	Canada McElheran et al. (2004) [ 34 ]; Kates et al. (2011) [ 41 ]; Paquette-Warren et al. (2006) [ 42 ]
Stepped approach	Family physicians or nurses attempt to address mental health and addiction issues before involving part-time onsite counselors, addiction specialists, child mental health professionals, or psychiatrists [41]	Canada Kates et al. (2011) [ 41 ]
Team-based model	Relies on the redefinition of roles for medical assistants and licensed practical nurses for groups of physicians at diverse practice sites (urban, rural, suburban) [38]	USA Misra-Hébert et al. (2018) [ 38 ]

### Organization and coordination of services in the co-located team-based model

To better illustrate how these approaches are operationalized within service organization and coordination, Table 3 presents, in each column: the principle orientation of services; practice context; team composition; which professional is the first entry point for accessing complementary services, service trajectories, and tools to support interprofessional collaboration within the team-based models. To improve the readability and comparability of the information, each column contains a list of possible options revealed during the results collation stage; more than one option could be selected in accordance with the information presented in the article. When the item did not present any information relating to the category presented in the column, the *not specified* box was checked.

**Table 3:** Organisation of team-based care models and supporting systems

Author, Year, Country	Principal orientation of services	Practice context (primary care)	Team composition	First point of entry	Service trajectories	Systems to support collaboration
Ashcroft et al. (2021) [32] Canada	☒Mental health ☐Chronic disease ☐Children and youth ☐Non specified	☒Family practice ☐Community primary care ☒Non specified	☒Family physician ☒Psychiatrist ☒Nurse practitioner ☒Registered nurse ☒Social worker ☐Care manager ☒Psychologist ☐Medical assistant ☒Dietician ☐Administrative staff ☒Pharmacist ☒Other mental health provider	☒Family physician ☐Nurse practitioner ☐Registered nurse ☐Medical assistant ☐Community mental health service ☐Screening tools for mental health problems ☐Any team member ☐Non specified	☐Predefined care plan ☐Predefined care trajectories ☒Non specified	☐Electronic medical record • Shared: ☐ Yes ☐ No ☐Patient registry • Shared: ☒ Yes ☐ No ☐Joint case consultation • Including the patient: ☐ Yes ☐ No ☐Joint care management • Including the patient: ☐ Yes ☐ No ☐ Team meetings ☐Others ☒Non specified
Blackmore et al. (2018) [28] USA	☒Mental health ☐Chronic disease ☐Children and youth ☐Non specified	☐Family practice ☐Community primary care ☒Non specified	☒Family physician ☒Psychiatrist ☐Nurse practitioner ☒Registered nurse ☒Social worker ☒Care manager ☐Psychologist ☐Medical assistant ☐Dietician ☐Administrative staff ☐Pharmacist ☐Other mental health provider	☒Family physician ☐Nurse practitioner ☒Registered nurse ☐Medical assistant ☐Community mental health service ☒Screening tools for mental health problems ☐Any team member ☐Non specified	☐Predefined care plan ☐Predefined care trajectories ☒Non specified	☒Electronic medical record • Shared: ☒ Yes ☐ No ☒Patient registry • Shared: ☒ Yes ☐ No ☒Joint case consultation • Including the patient: ☐ Yes ☐ No ☐Joint care management • Including the patient: ☐ Yes ☐ No ☐ Team meetings ☐Others ☐Non specified
Emerson et al. (2023) [40] USA	☒Mental health ☐Chronic disease ☐Children and youth ☐Non specified	☐Family practice ☐Community primary care ☐Non specified	☒Family physician ☐Psychiatrist ☒Nurse practitioner ☐Registered nurse ☐Social worker ☐Care manager ☐Psychologist ☒Medical assistant ☐Dietician ☐Administrative staff ☐Pharmacist ☐Other mental health provider	☒Family physician ☒Nurse practitioner ☐Registered nurse ☐Medical assistant ☐Community mental health service ☐Screening tools for mental health problems ☐Any team member ☐Non specified	☐Predefined care plan ☐Predefined care trajectories ☒Non specified	☒Electronic medical record • Shared: ☒ Yes ☐ No ☒Patient registry • Shared: ☒ Yes ☐ No ☐Joint case consultation • Including the patient: ☐ Yes ☐ No ☐Joint care management • Including the patient: ☐ Yes ☐ No ☒ Team meetings ☐Others ☐Non specified
Fitzpatrick et al. (2018) [33] Australia	☒Mental health ☐Chronic disease ☐Children and youth ☐Non specified	☒Family practice ☐Community primary care ☐Non specified	☒Family physician ☒Psychiatrist ☒Nurse practitioner ☐Registered nurse ☒Social worker ☐Care manager ☒Psychologist ☐Medical assistant ☐Dietician ☐Administrative staff ☐Pharmacist ☐Other mental health provider	☐Family physician ☐Nurse practitioner ☐Registered nurse ☐Medical assistant ☒Community mental health service ☐Screening tools for mental health problems ☐Any team member ☒Non specified	☐Predefined care plan ☐Predefined care trajectories ☒Non specified	☒Electronic medical record • Shared: ☐ Yes ☒ No ☐Patient registry • Shared: ☐ Yes ☐ No ☒Joint case consultation • Including the patient: ☐ Yes ☒ No ☒Joint care management • Including the patient: ☒ Yes ☐ No ☐ Team meetings ☐Others ☐Non specified
Kates et al. (2011) [41] Canada	☒Mental health ☒Chronic disease ☒Children and youth ☐Non specified	☒Family practice ☐Community primary care ☐Non specified	☒Family physician ☒Psychiatrist ☒Nurse practitioner ☒Registered nurse ☒Social worker ☐Care manager ☐Psychologist ☐Medical assistant ☒Dietician ☒Administrative staff ☒Pharmacist ☒Other mental health provider	☒Family physician ☒Nurse practitioner ☐Registered nurse ☐Medical assistant ☐Community mental health service ☒Screening tools for mental health problems ☐Any team member ☐Non specified	☐Predefined care plan ☐Predefined care trajectories ☒Non specified	☐Electronic medical record • Shared: ☐ Yes ☐ No ☐Patient registry • Shared: ☐ Yes ☐ No ☒Joint case consultation • Including the patient: ☐ Yes ☒ No ☒Joint care management • Including the patient: ☐ Yes ☒ No ☒ Team meetings ☐Others ☐Non specified
Lawson et al. (2012) [29] Canada	☐Mental health ☒Chronic disease ☒Children and youth ☐Non specified	☐Family practice ☒Community primary care ☐Non specified	☒Family physician ☐Psychiatrist ☒Nurse practitioner ☐Registered nurse ☐Social worker ☐Care manager ☐Psychologist ☐Medical assistant ☐Dietician ☐Administrative staff ☐Pharmacist ☐Other mental health provider	☐Family physician ☐Nurse practitioner ☐Registered nurse ☐Medical assistant ☐Community mental health service ☐Screening tools for mental health problems ☒Any team member ☐Non specified	☐Predefined care plan ☐Predefined care trajectories ☒Non specified	☐Electronic medical record • Shared: ☐ Yes ☐ No ☐Patient registry • Shared: ☐ Yes ☐ No ☐Joint case consultation • Including the patient: ☐ Yes ☐ No ☐Joint care management • Including the patient: ☐ Yes ☐ No ☐ Team meetings ☐Others ☒Non specified
Lyon & Slawson (2011) [37] USA	☐Mental health ☒Chronic disease ☐Children and youth ☐Non specified	☒Family practice ☐Community primary care ☐Non specified	☒Family physician ☐Psychiatrist ☐Nurse practitioner ☒Registered nurse ☐Social worker ☒Care manager ☐Psychologist ☒Medical assistant ☐Dietician ☐Administrative staff ☐Pharmacist ☐Other mental health provider	☒Family physician ☐Nurse practitioner ☐Registered nurse ☒Medical assistant ☐Community mental health service ☐Screening tools for mental health problems ☐Any team member ☐Non specified	☒Predefined care plan ☐Predefined care trajectories ☐Non specified	☒Electronic medical record • Shared: ☒ Yes ☐ No ☒Patient registry • Shared: ☒ Yes ☐ No ☐Joint case consultation • Including the patient: ☐ Yes ☐ No ☒Joint care management • Including the patient: ☐ Yes ☒ No ☒ Team meetings ☒Others ☐Non specified
McElheran et al. (2004) [34] Canada	☒Mental health ☐Chronic disease ☐Children and youth ☐Non specified	☒Family practice ☐Community primary care ☐Non specified	☒Family physician ☒Psychiatrist ☐Nurse practitioner ☒Registered nurse ☐Social worker ☐Care manager ☒Psychologist ☐Medical assistant ☐Dietician ☐Administrative staff ☐Pharmacist ☐Other mental health provider	☒Family physician ☐Nurse practitioner ☐Registered nurse ☐Medical assistant ☐Community mental health service ☐Screening tools for mental health problems ☐Any team member ☐Non specified	☐Predefined care plan ☐Predefined care trajectories ☒Non specified	☐Electronic medical record • Shared: ☐ Yes ☐ No ☐Patient registry • Shared: ☐ Yes ☐ No ☒Joint case consultation • Including the patient: ☒ Yes ☐ No ☐Joint care management • Including the patient: ☐ Yes ☐ No ☐ Team meetings ☐Others ☐Non specified
Misra-Hébert et al. (2018) [38] USA	☐Mental health ☒Chronic disease ☐Children and youth ☐Non specified	☐Family practice ☐Community primary care ☒Non specified	☒Family physician ☐Psychiatrist ☒Nurse practitioner ☐Registered nurse ☐Social worker ☒Care manager ☐Psychologist ☒Medical assistant ☐Dietician ☐Administrative staff ☐Pharmacist ☐Other mental health provider	☒Family physician ☐Nurse practitioner ☐Registered nurse ☐Medical assistant ☐Community mental health service ☐Screening tools for mental health problems ☐Any team member ☐Non specified	☐Predefined care plan ☐Predefined care trajectories ☒Non specified	☐Electronic medical record • Shared: ☐ Yes ☐ No ☐Patient registry • Shared: ☐ Yes ☐ No ☐Joint case consultation • Including the patient: ☐ Yes ☐ No ☐Joint care management • Including the patient: ☐ Yes ☐ No ☐ Team meetings ☒Others ☐Non specified
Moore et al. (2018) [35] USA	☒Mental health ☐Chronic disease ☒Children and youth ☐Non specified	☒Family practice ☐Community primary care practice ☐Non specified	☒Family physician ☒Psychiatrist ☒Nurse practitioner ☐Registered nurse ☐Social worker ☐Care manager ☒Psychologist ☐Medical assistant ☐Dietician ☐Administrative staff ☐Pharmacist ☐Other mental health provider	☐Family physician ☐Nurse practitioner ☐Registered nurse ☐Medical assistant ☐Community mental health service ☐Screening tools for mental health problems ☐Any team member ☒Non specified	☐Predefined care plan ☐Predefined care trajectories ☒Non specified	☒Electronic medical record • Shared: ☒ Yes ☐ No ☐Patient registry • Shared: ☐ Yes ☐ No ☐Joint case consultation • Including the patient: ☐ Yes ☐ No ☐Joint care management • Including the patient: ☐ Yes ☐ No ☒ Team meetings ☐Others ☐Non specified
Paquette-Warren et al. (2006) [42] Canada	☒Mental health ☒Chronic disease ☐Children and youth ☐Non specified	☐Family practice ☐Community primary care practice ☒Non specified	☒Family physician ☒Psychiatrist ☐Nurse practitioner ☐Registered nurse ☒Social worker ☐Care manager ☐Psychologist ☐Medical assistant ☒Dietician ☐Administrative staff ☐Pharmacist ☒Other mental health provider	☐Family physician ☐Nurse practitioner ☐Registered nurse ☐Medical assistant ☐Community mental health service ☐Screening tools for mental health problems ☐Any team member ☒Non specified	☐Predefined care plan ☐Predefined care trajectories ☒Non specified	☐Electronic medical record • Shared: ☐ Yes ☐ No ☐Patient registry • Shared: ☐ Yes ☐ No ☐Joint case consultation • Including the patient: ☐ Yes ☐ No ☐Joint care management • Including the patient: ☐ Yes ☐ No ☐ Team meetings ☐Others ☒Non specified
Roblin et al. (2011) [43] USA	☐Mental health ☐Chronic disease ☐Children and youth ☒Non specified	☐Family practice ☐Community primary care practice ☒Non specified	☒Family physician ☐Psychiatrist ☒Nurse practitioner ☒Registered nurse ☐Social worker ☐Care manager ☐Psychologist ☒Medical assistant ☐Dietician ☒Administrative staff ☐Pharmacist ☐Other mental health provider	☐Family physician ☐Nurse practitioner ☐Registered nurse ☐Medical assistant ☐Community mental health service ☐Screening tools for mental health problems ☐Any team member ☒Non specified	☐Predefined care plan ☐Predefined care trajectories ☒Non specified	☒Electronic medical record • Shared: ☒ Yes ☐ No ☐Patient registry • Shared: ☐ Yes ☐ No ☐Joint case consultation • Including the patient: ☐ Yes ☐ No ☐Joint care management • Including the patient: ☐ Yes ☐ No ☒ Team meetings ☒Others ☐Non specified
Rugkåsa et al. (2020) [30] Norway	☒Mental health ☐Chronic disease ☐Children and youth ☐Non specified	☒Family practice ☐Community primary care practice ☐Non specified	☒Family physician ☒Psychiatrist ☐Nurse practitioner ☐Registered nurse ☐Social worker ☐Care manager ☒Psychologist ☐Medical assistant ☐Dietician ☐Administrative staff ☐Pharmacist ☐Other mental health provider	☒Family physician ☐Nurse practitioner ☐Registered nurse ☐Medical assistant ☐Community mental health service ☐Screening tools for mental health problems ☐Any team member ☐Non specified	☐Predefined care plan ☐Predefined care trajectories ☒Non specified	☒Electronic medical record • Shared: ☒ Yes ☐ No ☒Patient registry • Shared: ☒ Yes ☐ No ☐Joint case consultation • Including the patient: ☐ Yes ☐ No ☐Joint care management • Including the patient: ☐ Yes ☐ No ☐ Team meetings ☐Others ☐Non specified
Sanchez & Adorno (2013) [36] USA	☒Mental health ☐Chronic disease ☐Children and youth ☐Non specified	☐Family practice ☐Community primary care practice ☒Non specified	☒Family physician ☒Psychiatrist ☐Nurse practitioner ☐Registered nurse ☒Social worker ☒Care manager ☐Psychologist ☐Medical assistant ☐Dietician ☐Administrative staff ☐Pharmacist ☐Other mental health provider	☒Family physician ☐Nurse practitioner ☐Registered nurse ☐Medical assistant ☐Community mental health service ☒Screening tools for mental health problems ☐Any team member ☐Non specified	☐Predefined care plan ☒Predefined care trajectories ☐Non specified	☐Electronic medical record • Shared: ☐ Yes ☐ No ☒Patient registry • Shared: ☒ Yes ☐ No ☒Joint case consultation • Including the patient: ☐ Yes ☒ No ☐Joint care management • Including the patient: ☐ Yes ☐ No ☐ Team meetings ☐Others ☐Non specified
Scanlon et al. (2008) [39] USA	☐Mental health ☒Chronic disease ☐Children and youth ☐Non specified	☐Family practice ☒Community primary care practice ☐Non specified	☒Family physician ☐Psychiatrist ☒Nurse practitioner ☒Registered nurse ☒Social worker ☒Care manager ☐Psychologist ☒Medical assistant ☐Dietician ☐Administrative staff ☐Pharmacist ☐Other mental health provider	☐Family physician ☐Nurse practitioner ☐Registered nurse ☐Medical assistant ☐Community mental health service ☐Screening tools for mental health problems ☐Any team member ☒Non specified	☒Predefined care plan ☐Predefined care trajectories ☐Non specified	☐Electronic medical record • Shared: ☐ Yes ☐ No ☒Patient registry • Shared: ☒ Yes ☐ No ☐Joint case consultation • Including the patient: ☐ Yes ☐ No ☐Joint care management • Including the patient: ☐ Yes ☐ No ☐ Team meetings ☐Others ☐Non specified

Although all models were situated in a primary care context, seven of these (7/15; 46,7%) were implemented in family medical practices similar to a FMG or a FHT. Two models were community-oriented, for example, the community primary healthcare in Nova Scotia, which provided health promotion activities and programs in a family medical practice but also in other community settings (e.g., outreach programs) [29]. The most common service orientation was for people with mental health conditions (10/15; 66,7%) and chronic disease (6/15; 40%). Some models provided services directed to young people or older adults. In mental health, teams are mainly staffed by family physician, nurses, psychiatrists, psychologists and social workers [28, 30, 32–36]. For chronic diseases, teams generally comprise family physicians, nurses (RN, NP or nurse coordinator) [29, 37–39] and medical assistants [29, 37–40]. An exception is Scanlon and colleagues’ [39] model, which includes a social worker. Only the three Ontario (Canada) FHT models integrated both mental health professionals and dieticians [32, 41, 42].

Some team-based models (5/15; 33,3%) included a care manager, this role most frequently being filled by a nurse [37–39] or a social worker [36]. Care managers facilitate patient engagement and education [28, 36, 41], provide proactive follow-up [28, 36, 37] and coordinate care [28, 36, 37, 41].

Regarding which professional is the entry point for accessing complementary services, the FP was the professional most cited (9/15; 60%), followed by nurses (3/15; 20%). These professionals evaluate patients’ needs and refer them to other professionals, and, in three (3/15;20%) team-based models, they also use a screening tool to detect mental health problems. In the case of Kates’ study [41], dieticians also used screening tools with individuals with diabetes to screen for depression. The trajectory of patients following this initial assessment is infrequently specified in the articles; in only three studies (3/15; 20%) was the existence of a predefined service trajectory or care plan for patients specified.

Regarding the collaboration tools used within the team, the most frequent tools identified include team meetings (5/15, 33,3%), shared electronic medical record or patient registry (8/15; 53,3%), and joint care planning, consultation or management (6/15; 40%). Only two studies explicitly named the patient as an integral part of care planning [33, 34].

The management of the team-based models was described in six studies (6/15; 40%) [33, 36, 40–43]. They mostly describe how the management roles are shared between professionals or organizations involved, sometimes with a description of these roles, for example: a FP can run the clinic [33]; diverse sites can have one central management team [42]; each practice can be autonomous in decisions [43].

### Contexts of emergence of the co-located team-based care models

Consistent with our objectives, we summarized the data regarding contexts to better understand the emerging context of the team-based model and the type of support provided. These data are inconsistently reported across the articles. Nevertheless, we have been able to capture the models’ main goals, the sector’s characteristic in which they have been implemented, if they have been implemented in more than one practice site, and the type of support provided.

The goals of the team-based primary care models studied have been explicitly reported in 7/15 (46,7%) studies. The main goals included: improve access to complementary services, especially mental health services [32, 40–42]; improve quality of care, clinical outcomes and patient experience [28, 37, 41]; improve skills of family physician, especially in mental health [34, 41, 42]; use resources more efficiently [41].

The characteristics of the sector in which the model has emerged have been provided by all studies with, however, considerable variability regarding the details. The models were implemented in an urban/suburban area (8/15; 53,3%) [28, 34, 35, 37, 40–43], in a rural area (3/15; 20%) [29, 33, 39] or in both (1/15; 6,7%) [32]. Some initiatives (3/15; 20%) specifically focused on districts with vulnerable or marginalized populations (e.g., low-income) [35, 36, 39]. Several sectors (4/15; 26,7%) were characterized by poor access to mental health services, resulting in under-treatment of these conditions [30, 33, 34, 41]. In several sectors (3/15; 20%), the family physician’s role was identified as important in providing mental health care [30, 33, 41]. A shortage of specialists was also highlighted in a few sectors (2/15; 13,3%) [33, 34]. Only one model specifically addressed chronic medical conditions; the authors reported that most patients in their area were underserved, and performance needed to be improved [37].

Some models (6/15, 40%) have been developed in only one practice [29, 30, 33, 36, 37, 40]. Others have been deployed locally in several sites; either in one area (8/15; 53,3%) [28, 34, 35, 38, 39, 41–43] (e.g., Hamilton, Ontario [41, 42]) or within a single province/state (1/15; 6,7%) [32].

The type of support documented in the article was limited to the type of financial support provided. When reported, the authors provided varying levels of detail regarding funding. The type of funding received varied significantly. It was possible to discriminate the type of physician’s payment and the source of the funding for the complementary services (federal, provincial, private, municipal). The type of physicians’ payment was indicated in 3/15 (20%) articles, all situated in Canada [29, 32, 41]. Physicians within these team-based models were primarily compensated through alternative funding arrangements, with capitation-based funding being the most common approach [32, 41]. Regarding complementary services in the US, Canada and Australia, funding came from federal public funds (3/15; 20%) [28, 39, 40], private foundations (2/15; 13,3%) [36, 43], provincial or state (2/15; 13,3%) [33, 41], or a joint federal and provincial initiative [34]. One model was funded by a local public program (*Mental Health and Nutrition Program* in Hamilton, Canada) [42]. In another model located in Norway, physicians were paid in three different ways (capitation based, fee-for-service, co-payments by patients). In this model, although mental health professionals provided a free service to patients, these services could not be funded if not provided within these professionals’ own employment structure [30].

### Impacts on population, providers and healthcare costs

Study objectives, design, participants, and main results are presented in Table 4 to present the information related to the impacts of the team-based primary care models, and the key factors that influenced these impacts. The results regarding impacts were classified according to their target: patients, providers, and cost-effectiveness. The impact of team-based models on patients have been reported in 14/15 (93,3%) studies, while 7/15 (46,7%) studies reported effects on providers. The main effects on patients include: improved access and reduced stigma, especially for individuals with mental health problems [32, 41, 42]; enhanced chronic disease management [29]; better treatment adherence and follow-up [28, 33, 35]; better relationship with FP [32–34]; and improvement in symptoms or functioning [28, 40, 41]. Positive impacts on providers include: upskilling [33, 34, 42]; better job satisfaction [33, 37]; and redistribution of workloads [37].

**Table 4:** Study objectives, design, participants and main results

Author, Year, Title, Country	Study objectives	Design, Participants	Main Results
Ashcroft et al. (2021) [ 32 ] *Patient perspectives on quality of care for depression and anxiety in primary health care teams: A qualitative study* Canada	To understand patients’ perspectives on the quality of care that they received for common mental disorders from Ontario's Family Health Teams	Qualitative (constructivist grounded theory) 40 patients from 38 Family Health Teams: • Focus groups: 31 • Individual interviews: 9	**Patients** • Variations existed within and across FHTs regarding the types of mental health providers and therapeutic modalities used in therapy • Patients highlighted the importance of meeting diverse needs [understanding impact of socio-economic status on patients’ mental health (e.g., culture, gender)] • Benefits of the program: o improved access to multiple mental health services; o reduced stigmatisation; o having a strong relationship with a physician helped enhance the shared decision-making process • Areas for improvement: o length of appointments with physicians considered inadequate; o caps on the number of therapy sessions viewed as problematic; o all participants expressed a strong desire to have mental health screening and assessment routinely integrated into their primary care; o difficulty with coordination between off-sites psychiatrists; o follow-up needs to be improved
Blackmore et al. (2018) [ 28 ] *Comparison of collaborative care and colocation treatment for patients with clinically significant depression symptoms in primary care* USA	Cross-sectional prospective comparison of co-located care and the Collaborative Care Model (CoCM) for patients reporting clinically significant depression symptoms in primary care to assess differences in clinical outcomes	Quantitative (case comparison) 240 patients (122 at colocation sites; 118 at the CoCM sites) • PHQ-9 scores at baseline and 12-week follow-up	**Patients** • Benefits of the addition of a care manager (in CoCM): o greater reduction in depression symptoms; o more contacts with social worker; o facilitate communication, treatment adherence and self-management; o improved follow up • CoCM emphasized problem solving therapy and cognitive-behavioral therapy by social workers and behavioral activation and self-management education by care managers • CoCM appears to offer a more effective model for providing treatment of depression symptoms in primary care, compared with the colocation model
Emerson et al., (2023) [ 40 ] *Improving integrated mental health care through an advanced practice registered nurse-led program: Challenges and successes* USA	The objective of this case study was to describe the implementation of an advanced practice registered nurse (APRN)– led integrated behavioral health care program, including the challenges, barriers, and successes encountered in the first 9 months	Quantitative 14 FP • Survey 86 patients • PHQ-9 and GAD-7	**Patients** • Improvement in anxiety and depression symptoms • Improvement of accessibility of behavioral health provider (BHP) services • Overall satisfaction with BHP services
Fitzpatrick et al. (2018) [ 33 ] *Coordinating mental and physical health care in rural Australia: An integrated model for primary care settings* Australia	To examine integrated care in an established mainstream rural service developed without additional pilot funding or changes in systems or incentives	Qualitative and quantitative (multiple methods case study) 16 health care providers and other staff associated with the GP Clinic [ community mental health team members (n = 11), general practitioners (n = 2), visiting and consultant psychiatrists (n = 2), and practice managers (n = 1)]: • Individual interview 65 patients: • Clinical data collected from service records	**Patients** • Developed longer-term relationships with the FP • Improved access to physical health care (reduced stigma, shorter waiting list, help with booking systems, more empathic physician) • Improved continuity of care • Improved physical health care • Improved follow-up **Providers** • Improved care planning and treatment through improved team communication • Upskilling of local clinical staff • Increased trust between FP and psychiatrist • Better quality of working life, and job satisfaction • Time and capacity for the FP to provide care to those with complex needs and remuneration were described as important incentives • The precariousness of Medicare funding was a potential barrier to its ongoing financial viability for the FP • Successful operation of the FP clinic was dependent on current staffing levels **Cost-effectiveness** • Organisational and cost-saving benefits by ensuring more efficient practices
Kates et al. (2011) [ 41 ] *Integrating mental health services within primary care settings: the Hamilton Family Health Team* Canada	This article reviews the evolution of the program and the changes made by practices with key lessons learnt	No methodology is presented; only a self-evaluation of the clinic	**Patients** • Improvements in symptoms and/or functioning • Better access to mental health care, particularly for: o individuals who come from diverse ethnocultural groups; o children; o seniors • Reduced waiting times for an initial assessment • Earlier detection and treatment of mental health and addiction problems • Less stigmatisation • Better care coordination between primary, secondary, and tertiary mental health services **Key enablers of success** • Having a clear vision and direction for the program from the outset • Customizing the HFHT-MHP model to each clinic’s patient population, staff, and resources within the clear guidelines laid down by the program management team • Care is shared by the mental health and primary providers, according to their respective skills, comfort, and availability • Assistance from a management team in coordinating the practice activities and resolving problems • Family physicians willing to participate in program activities, particularly in discussing cases with any of their mental health team colleagues
Lawson et al. (2012) [ 29 ] *Using quality indicators to evaluate the effect of implementing an enhanced collaborative care model among a community, primary healthcare practice population* Canada	To evaluate the effect of an enhanced collaborative care model, (which includes family physicians, nurse practitioner team member), on the quality of healthcare delivery in a community primary health care practice	Quantitative (retrospective observational design) 392 patients’ charts were audited (197 pre-period; 195 post-period)	**Patients** • Better quality healthcare delivery • Enhanced chronic disease management **Facilitators**: • Strong health authority and physician support • Skills and working style of the NP and early proactive team-building activities, which helped to increase awareness of the NP role • Remuneration of physician
Lyon & Slawson (2011) [ 37 ] *An organized approach to chronic disease care* USA	This article describes the authors’ experience, focusing primarily on how teams were developed and used to improve chronic disease care	No methodology, only implementation is described	**Patients** • Joint care plan management resulted in improved patient engagement • Improvement of preventive care • Not all patient responded to this team approach **Providers** • Greater work satisfaction • Redistribution of workloads
McElheran et al. (2004) [ 34 ] *Shared mental health care: the Calgary model* Canada	To evaluate a time-limited demonstration project (The Shared Mental Health Care, Calgary Model)	Program evaluation 143 patients • Patient satisfaction questionnaire • Patient enablement questionnaire • SF-36 Health Survey Physician (final number unknown) • Physician enablement questionnaire	**Patients** • Better management and self management of patients’ mental health (improved self efficacy) • Felt getting better • Better relationship with FP **Providers** • Upskilling of FP, but still need development and maintenance of these new skills • Feeling more effective in managing health mental issues (more confident and more support) • Less outside referral
Misra-Hébert et al. (2018) [ 38 ] *Implementing team-based primary care models: a mixed-methods comparative case study in a large, integrated health care system* USA	The goal was to identify characteristics in the practice settings that contributed to uptake of these new models Models: In team-care (TC), two medical assistants or licensed practical nurses assigned to one physician performed team documentation (scribing) and administrative work. In a second model, or modified team-care (MTC), an additional medical assistant assigned to a group of three physicians (for a total of 4 medical assistants per team) helped with clinical workflow or administrative work	Comparative case study of 9 primary care practices where physicians implemented either (or both) of these models using a convergent mixed methods approach Practice observations and informal interviews at 9 sites, follow-up observations at 7 sites 19 FP (13 practicing in team-based models, 5 in usual care at the same sites, 1 was a key informant), 18 medical assistants/licensed practical nurses, 8 nurse care coordinators, and 30 patients • Individual interview 276 employees (FP, NP, physician assistants, medical assistant, licensed practical nurse, nurses, and administrative staff) (response rate 35%) at time 0 and 238 at time 1 (response rate 33%) • Survey Quality scores of provider’s performances at 9 sites (e.g., preventive care measures, chronic disease measures)	**Patients** • Overall acceptance of team-based care • Satisfied with the new roles and involvement of a new professional in all practice sites • Shorter wait times **Providers** • Uptake of the new team-based models as originally intended, can be influenced by: o a practice ability to respond to change o a flexible workflow as related to team roles o strength of local leadership o stable staffing • Practices with high uptake of new models performed better overall on quality metrics (especially on preventive care and diabetes disease control)
Moore et al. (2018) [ 35 ] *Practice procedures in models of primary care collaboration for children with ADHD* USA	To examine the impact of two co-located models of primary care (with differing levels of behavioral health integration) on American Academy of Pediatrics guideline adherence for the assessment and treatment of Attention-deficit/hyperactivity disorder (ADHD)	Retrospective chart review 149 patients’ charts (74 integrated and 75 co-located practice charts)	**Patients** • No difference between practices in diagnostic validity • Engagement with a psychologist on site in the integrated setting led to: o improved adherence to ADHD guidelines o improved follow-up (engagement of patient) o improved patient experience
Paquette-Warren et al. (2006) [ 42 ] *What do practitioners think? A qualitative study of a shared care mental health and nutrition primary care program* Canada	To obtain the perspective of health care practitioners on the structure, implementation, and functioning of a shared care model. To answer the following questions: (1) What are the goals of the program from the provider perspective? (2) What are the strengths of the shared care program?; (3) What challenges do the providers face in running the program?; and (4) How efficacious is a shared care model for a variety of populations?	Qualitative (Qualitative descriptive) 8 FP, 7 psychiatrists, 13 mental health counsellors, 4 registered dieticians, 21 practitioners • Focus group	**Patients** • According to the providers: o improved accessibility and continuity of care; o improved patient experience, reduced stigma; o patient empowerment; o patients benefiting the most from the program are: patients with institutional barriers, family problems, general psychiatric ailments, severe mental health and physical problems (e.g., diabetes, lipidemia, gastrointestinal issues, patients with low socio-economic status, elderly, and ethnic groups) **Providers** • Overall satisfaction with the program’s structure, implementation, and functioning and view it as a critical service in their community • Benefits of the program according to the providers: o improved knowledge and capability of practitioners o access to pertinent patient information o facilitate referrals to tertiary care services o less use of emergency and hospital services • Shared-care model: o its enactment varies by providers and practices o key features influencing its application: communication, availability of team members, physical space to work simultaneously, individual skills and comfort, working relationships, and family physicians’ perspective of shared care o time and spaces challenges, because of lack of adequate funding
Roblin et al. (2011) [ 43 ] *An evaluation of the influence of primary care team functioning on the health of Medicare beneficiaries* USA	To evaluate the association of primary care teams functioning as perceived by practitioners and support staff on 14 primary care teams with health status reported by Medicare beneficiaries at 2 years following a baseline assessment in a group model managed care organization (MCO)	Quantitative (survey) Primary care teams practitioners and support staff on 14 primary care teams • 190 completed the survey in mid-2000 (83% response rate) • 239 completed the survey in mid-2002 (91% response rate) 991 Medicare beneficiaries • SF-36 survey	**Patients** • Medicare beneficiaries with one or more major morbidities who were assigned to relatively high functioning primary care teams had significantly better physical and mental health 2 years later compared with Medicare beneficiaries assigned to relatively low functioning primary care teams **Providers** • High functioning primary care teams depend on perceptions of team orientation, task management, and intrateam interactions reported by practitioners and support staff
Rugkåsa et al. (2020) [ 30 ] *Collaborative care for mental health: a qualitative study of the experiences of patients and health professionals* Norway	To identify, from the perspectives of patients and health professionals, the collaborative care model’s advantages, and disadvantages as well as its enablers and barriers	Qualitative (qualitative descriptive) 6 community mental health centres specialists, 7 FP, 11 patients • Individual interviews	**Patients** • Improved accessibility of mental health services, through co-location of general practitioner and mental health specialists • Less stigma in consulting at the general practitioner rather than at the community mental health centre **Providers** • Improved patient-centred case collaboration between general practitioners and mental health specialists • Upskilling of FP to deal with mental health problem and of mental health practitioners to deal with co-morbidities • Early intervention prevents conditions from deteriorating • Improvement of referral practices • Different modes of working can impede practices and learning (especially when they had insufficient knowledge of each other) • Funding was perceived as the most important barrier to collaborative care
Sanchez & Adorno (2013) [ 36 ] *"It's like being a well-loved child": Reflections from a collaborative care team* USA	The purpose of this case study is to examine a collaborative care model of service delivery for the treatment of depression with a low-income, uninsured adult population in a primary care setting. We explore how a collaborative care model of service delivery works in this setting	Qualitative (single case study) 3 interdisciplinary team members (clinical social worker as care manager; FP; consulting psychiatrist), and the clinic’s director of social services • Individual interviews	**Providers** • Usefulness of the PHQ-9 as a tool for tracking and for engagement of patient and to provide feedback of their improvement • Use of a physician champion to facilitate implementation and acceptation of the program to physicians, especially the social worker role as a care manager
Scanlon et al., 2008 [ 39 ] Financial and clinical impact of team-based treatment for Medicaid enrollees with diabetes in a federally qualified health center USA	The purpose of this study was to determine whether multidisciplinary team-based care guided by the chronic care model can reduce medical payments and improve quality for Medicaid enrollees with diabetes	Economic evaluation (difference-in-difference analysis) 193 CareSouth patients and 193 control patients • Financial analysis	**Patients** • Improvement in diabetes outcomes for patients, but no comparison to control group (data not available) • Improvements are not associated with higher drug cost, so lifestyle management should explain the improvement **Cost-effectiveness** • No significant short terms savings compared to the control group, except for hospital-based outpatient visits

Some key facilitators of these benefits include: the addition of a care manager [28, 35]; strong leadership and support from the management team [29, 38, 41]; physician’s adherence to the model [29, 41]; stable staffing [33, 38]; individual skills and comfort with new or modified professional roles [29, 41, 42]; and shared vision of the model [41, 43]. The lack of adequate funding is the main barrier reported by the studies [29, 30, 33, 42].

Cost-effectiveness has been documented in 2/15 (13,3%) studies. However, although some organisational and cost savings benefits by ensuring more efficient practices have been reported by Fitzpatrick and colleagues [33] and Scanlon and colleagues’ study [39], they do not show significant changes in cost or use of healthcare resources.

## Discussion

### Team composition, role optimization and organisation of services

The results indicate that team-based care models have been operationalized in various ways within primary care environments. The models reviewed were predominantly implemented in the United States and Canada, which raises the question regarding the contextual elements that might be associated with the implementation of these kinds of models.

The composition of teams in the reviewed models is diverse, encompassing various professionals in addition to family physicians. With minor exception, the models tend to focus either on the provision of mental health services, on chronic disease management, but rarely on both. Team composition appears to be associated with the nature of the services offered. Evidence suggests that the addition of a broader range of health care providers (e.g., pharmacists, nurse) expands the array of services, and that it improves health outcomes [44]. Our findings suggest that it is also important to consider the type of professionals involved, as the absence of certain professionals in the team may have an impact on the quality of care, the nature of services provided and the optimal use of roles. For example, Vader and colleagues [45] reported that the absence of a physiotherapist within the team limited the quality of services for patients with chronic low back pain. The integration of dieticians or rehabilitation specialists (e.g., physiotherapists, occupational therapists) also appears to be very limited in the models examined. This finding is of concern particularly regarding chronic diseases given the important role that these professionals play in improving patients' day-to-day health and functioning, promoting prevention, and reducing the use of healthcare system resources (e.g., fewer medical visits and hospitalizations) [46–48]. Although several models that integrate rehabilitation in primary care exist, most of these are designed for specific populations (e.g., older adults, individuals with disabilities) or in community-based settings [49].

The organisation of the team-based models included in this review emphasizes the role of family physicians as the primary entry point and assessors of patients' needs, as expected in a PMH model. Nevertheless, a few models relied more on nurses (RN or NP depending on the model) to perform these roles, which suggests a greater use of the nurses’ expertise in the team. More generally, some models illustrate the effective integration and versatility of nurses (NPs or RNs), who occupy clinical, coordination and educational roles, as many of the care managers reported were nurses. This finding is consistent with recent studies highlighting both NPs’ essential roles in primary care (e.g., managing patients, improving access to care, dealing with complex health care issues) and the need to improve their integration in primary care teams [50–52].

Regarding collaboration, most team-based models used tools (e.g., team meetings, shared electronic records) that facilitated communication and coordination among team members. Support from managers appears to be essential for facilitating collaboration. Our findings are consistent with the findings of Wranik and colleagues’ [44] systematic review, which revealed strong evidence that co-location, shared tools and appropriate leadership are important factors for collaboration. However, the inclusion of patients as integral participants in care planning appeared to be missing from the models reviewed, highlighting the potential for greater patient engagement. This engagement is recognized as an essential aspect of team-based care, especially in PMH [53].

Our review revealed a wide range of model of care approaches underpinning team-based care, which might reflect the flexibility necessary to cater to different healthcare contexts and patient populations. It is important to understand how these approaches influence the organization and delivery of care. As noted, given that most of the studies reviewed were conducted in North America, some prudence is required regarding their applicability within other countries.

### Tailoring the models to the specific characteristics and needs of a sector

The models identified primarily target individuals with mental health conditions and chronic diseases. The emphasis on these populations underscores the need for integrated and comprehensive care for individuals with co-morbidities, whether mental health problems or chronic diseases. This point also highlights the necessity to integrate social and health services within the same location, to better respond to these complex healthcare needs [54, 55]. Some initiatives specifically target districts with vulnerable or marginalized populations, for example, low-income communities, highlighting the models' required adaptability to address health disparities. This orientation is consistent with the fifth aim of primary care transformation [5].

No universal socio-demographic or socio-professional characteristics in favor of the development of these team-based models emerged in our review. Rather, what appears to be important is the careful study of the specific characteristics and needs of a sector. Nevertheless, the results permit the identification of elements to consider when implementing a team-based model: the sector (urban, suburban, rural); the population's need for services (e.g., mental health, chronic diseases); the type and ratio of professionals available in the sector, and consequently, the professionals required. The goal is to match the supply, access to, and quality of services in a sector and the population's need for services (type of physical and mental health problems). These elements reinforce Tomoaia-Cotisel and colleagues’ [21] highlighting of important contextual factors at multiple levels (level 1: external environment; level 2: larger organization; level 3: practice), diverse perspectives and data source, and the interactions between contextual factors and both the process and outcome of studies.

### Limited integration of population health outcomes, patients’ and providers’ experience and healthcare costs

Our review provides some insights into the outcomes of team-based care models, which encompass a range of benefits for patients and providers. Although some prudence in interpretation is required given that we did not evaluate the methodology of the studies, the models examined appear to generally satisfy the expectations of the overarching framework of a high-performing team-based primary care model at patient and provider levels [3, 6, 56]. On the other hand, the studies revealed few outcomes regarding prevention and patient self-management, even though these are important goals of primary care reform [3].

Only two articles provide results, which are inconclusive, regarding cost-effectiveness [33, 39]. These findings are consistent with those of other studies that have examined various forms of physician and complementary services financing under different models in North America and their cost-effectiveness [17, 57]. Furthermore, our results suggest that there has been little integration of qualitative data regarding patients’ experiences with the cost effectiveness data.

Several studies in our review discussed factors that facilitated or hindered positive outcomes for patients and providers (e.g., inadequate funding, presence of care managers, physician adherence, stable staffing, individual skills, shared vision). These results are consistent with Rawlinson and colleagues’ [20], analysis, in which these barriers and facilitators were classified into four categories: system (e.g., financing, supportive policies and government), organizational (e.g., co-location, effective leadership, shared data system, staffing), inter-individual (e.g., communication, common goals), individual (e.g., resistance to change, intention to collaborate).

Despite inadequate funding being a common barrier in the implementation of the team-based models, limited descriptions of model funding were provided in the reviewed studies. How funds are allocated, the modalities of professional payment, or reimbursement for services remain unclear, as does the distinction between funding for physicians and supplementary services. Funding arrangements appear to be complex as they involve multiple stakeholders at federal, provincial/state, or municipal levels.

### Implications for research

Regarding research implications, it is essential to integrate a multi-level analysis to consider elements from the external environment/system (macro level) such as population needs, funding or government policy; larger organization (meso level) such as co-location, leadership or type of provider available; and at the practice/individual (micro level), for example, communication, individual skills and readiness for change or role optimization, to gain a comprehensive understanding of these models. The importance of integrating economic and quantitative evaluations with qualitative aspects is also recognized. Finally, it will be important to identify the most relevant indicators for assessing a model’s performance.

### Limitations of the study

The extensive variability in the concepts used, their meanings, and the diversity of implemented models has made the process of selecting and analyzing articles complex. Although the Peek and National Integration Academy Council [58] has attempted to resolve this issue with a lexicon, this tool appears to be seldom used, and was not referred to in our reviewed studies. In our efforts to maintain rigour, several types of models were not included in this review. Models that rely on the integration of coordinated intervention programs were especially numerous. The TEAMcare, IMPACT, or COMPASS programs are examples of this type of program that target the treatment of depressive symptoms and certain chronic conditions [59]. Instead, we have opted for team-based models that rely on the integration of professionals to expand the range of primary care services and improve access without imposing special conditions on access to those services. Furthermore, as our primary focus was on service organization and the context of model implementation, numerous models that could have met the desired model type were excluded. Despite these limitations, the selected articles provided a range of pertinent details. Future research should seek a more comprehensive documentation of these elements and their integration into the model analysis.

## Conclusion

Studies rarely provide an overarching view that permits an understanding of the specific contexts, service organization, impact on the population, providers and healthcare costs, and the broader context of implementation. Therefore, it is difficult to identify universal guidelines for the functioning of efficient models. This point highlights the inherent complexity of operationalizing these models. It also underscores the importance of tailoring them to the specific needs and characteristics of a population in a given area, and reflecting on which professionals should be included in the team to truly meet those needs. In this sense, our results permit greater reflection on the deployment of professional role optimization within these co-located team-based models. A collaborative approach with the various stakeholders, including patients, involved in service organization appears to be essential to achieve this outcome.

## Data Availability

All data generated or analysed during this study are included in this published article.

## Abbreviations

FHT: Family health teams
FMG: Family medecine group
FP: Family physician
NP: Nurse practitioner
PMH: Patient’s medical home
RN: Registered nurse

## References

[1] World Health Organization. Primary care. World Health Organization n.d. https://www.who.int/teams/integrated-health-services/clinical-services-and-systems/primary-care. Accessed 11 Oct 2023).

[2] World Health Organization. Access to health services “Integrated people-centred health services” PHC-oriented models of care. World Health Organ 2024. https://www.emro.who.int/uhc-health-systems/access-health-services/phc-oriented-models-of-care.html. Accessed 20 May 2024.

[3] Aggarwal M, Hutchison B. Toward a primary care strategy for Canada. Ottawa (CA): Canadian Foundation for Healthcare Improvement; 2012.

[4] Canadian Institutes of Health Research. Taking the pulse: A snapshot of Canadian health care, 2023 2023. https://www.cihi.ca/en/taking-the-pulse-a-snapshot-of-canadian-health-care-2023/88-of-canadians-have-a-regular-health. Accessed 13 May 2024.

[5] Nundy S, Cooper LA, Mate KS. The quintuple aim for health care improvement: a new imperative to advance health equity. J Am Med Assoc. 2022;327:521–2. 10.1001/jama.2021.25181. 10.1001/jama.2021.25181 35061006

[6] Bodenheimer T, Sinsky C. From triple to quadruple aim: care of the patient requires care of the provider. Ann Fam Med. 2014;12:573–6. 10.1370/afm.1713. 25384822 10.1370/afm.1713 PMC4226781

[7] Government of Canada. ARCHIVED- A 10-year plan to strengthen health care 2004. https://www.canada.ca/en/health-canada/services/health-care-system/health-care-system-delivery/federal-provincial-territorial-collaboration/first-ministers-meeting-year-plan-2004/10-year-plan-strengthen-health-care.html. Accessed 11 Oct 2023.

[8] Collins C, Heuson DL, Munger R, Wade T. Evolving models of behavioral health integration in primary care. New York (NY): Milbank Memorial Fund; 2010.

[9] Nelson S, Turnbull J, Bainbridge L, Caulfield T, Hudon G, Kendel D, et al. Optimizing scopes of practice: New models of care for a new health care system. Ottawa (CA): Canadian Academy of Health Sciences; 2014.

[10] Bourgeault IL, Merritt K. Deploying and managing health human resources. Palgrave Int. Handb. Healthc. Policy Gov., London: Palgrave Macmillan UK; 2015, p. 308–24.

[11] Reeves S, Pelone F, Harrison R, Goldman J, Zwarenstein M. Interprofessional collaboration to improve professional practice and healthcare outcomes. Cochrane Database Syst Rev. 2017;6:1–38. 10.1002/14651858.CD000072.pub3. 10.1002/14651858.CD000072.pub3 PMC6481564 28639262

[12] College of Family Physicians of Canada. A new vision for Canada: family practice—the patient’s medical home 2019. Mississauga, ON: College of Family Physicians of Canada; 2019.

[13] Sibbald SL, Misra V, daSilva M, Licskai C. A framework to support the progressive implementation of integrated team-based care for the management of COPD: a collective case study. BMC Health Serv Res. 2022;22:1–11. 10.1186/s12913-022-07785-x. 35354444 10.1186/s12913-022-07785-x PMC8966237

[14] Beck A, Boggs JM, Alem A, Coleman KJ, Rossom RC, Neely C, et al. Large-scale implementation of collaborative care management for depression and diabetes and/or cardiovascular disease. J Am Board Fam Med. 2018;31:702–11. 10.3122/jabfm.2018.05.170102. 30201666 10.3122/jabfm.2018.05.170102

[15] Plourde A. Bilan des groupes de médecine de famille après 20 ans d’existence: Un modèle à revoir en profondeur. Montréal: Inst Rech Inf Socioéconomiques; 2022. p. 1–25. https://iris-recherche.qc.ca/apropos-iris/nous-joindre/.

[16] Asarnow JR, Rozenman M, Wiblin J, Zeltzer L. Integrated medical-behavioral care compared with usual primary care for child and adolescent behavorial health: A meta-analysis. JAMA Pediatr. 2015;169:929–37. 10.1001/jamapediatrics.2015.1141. 26259143 10.1001/jamapediatrics.2015.1141

[17] Carter R, Riverin B, Levesque J-F, Gariepy G, Quesnel-Vallée A. The impact of primary care reform on health system performance in Canada: A systematic review. BMC Health Serv Res. 2016;16:1–11. 10.1186/s12913-016-1571-7. 27475057 10.1186/s12913-016-1571-7 PMC4967507

[18] McManus LS, Dominguez-Cancino KA, Stanek MK, Leyva-Moral JM, Bravo-Tare CE, Rivera-Lozada O, et al. The patient-centered medical home as an intervention strategy for diabetes mellitus: a systematic review of the litterature. Curr Diabetes Rev. 2021;17:317–31. 10.2174/1573399816666201123103835. 33231158 10.2174/1573399816666201123103835

[19] Norful AA, Swords K, Marichal M, Hwayoung C, Poghosyan L. Nurse practitioner-physician comanagement of primary care patients: The promise of a new delivery care model to improve quality of care. Health Care Manage Rev. 2019;44:235–45. 10.1097/HMR.0000000000000161. 28445324 10.1097/HMR.0000000000000161 PMC5656564

[20] Rawlinson C, Carron T, Cohidon C, C A, Hong QN, Pluye P, et al. An overview of reviews on interprofessional collaboration in primary care: Barriers and facilitators. Int J Integr Care 2021;21:1–25. 10.5334/ijic.5589. 10.5334/ijic.5589 PMC8231480 34220396

[21] Tomoaia-Cotisel A, Scammon DL, Waitzman NJ, Cronholm PF, Halladay JR, Driscoll DL, et al. Context matters: the experience of 14 research teams in systematically reporting contextual factors important for practice change. Ann Fam Med. 2013;11:S115–23. 10.1370/afm.1549. 23690380 10.1370/afm.1549 PMC3707255

[22] Longhini J, Canzan F, Mezzalira E, Saiani L, Ambrosi E. Organisational models in primary health care to manage chronic conditions: a scoping review. Health Soc Care Community. 2022;30:565–88. 10.1111/hsc.13611. 10.1111/hsc.13611 34672051

[23] Arksey H, O’Malley L. Scoping studies: towards a methodological framework. Int J Soc Res Methodol. 2005;8:19–32. 10.1080/1364557032000119616. 10.1080/1364557032000119616

[24] Levac D, Colquhoun H, O’Brien KK. Scoping studies: advancing the methodology. Implement Sci. 2010;5:1–9. 10.1186/1748-5908-5-69. 20854677 10.1186/1748-5908-5-69 PMC2954944

[25] Reeves, S, Lewin, S, Espin, S, Zwarenstein, M. A conceptual framework for interprofessional teamwork. Interprofessional Teamwork Health Soc. Care. Oxford: Blackwell Publishing Ltd.; 2010, p. 57–76.

[26] Cancer Care Ontario. 10 tools for implementing new models of care: a guide to change management. Cancer Care Ontario; 2017.

[27] World Health Organization, United Nations Children’s Fund (UNICEF). Operational framework for primary health care: Transforming vision into action. World Health Organization; 2020.

[28] Blackmore MA, Carleton KE, Ricketts SM, Patel UB, Stein D, Mallow A, et al. Comparison of collaborative care and colocation treatment for patients with clinically significant depression symptoms in primary care. Psychiatr Serv. 2018;69:1184–7. 10.1176/appi.ps.201700569. 30152273 10.1176/appi.ps.201700569

[29] Lawson B, Dicks D, Macdonald L, Burge F. Using quality indicators to evaluate the effect of implementing an enhanced collaborative care model among a community, primary healthcare practice population. Nurs Leadersh. 2012;25:28–42. 10.12927/cjnl.2013.23057. 10.12927/cjnl.2013.23057 23010918

[30] Rugkåsa J, Tveit OG, Berteig J, Hussain A, Ruud T. Collaborative care for mental health: a qualitative study of the experiences of patients and health professionals. BMC Health Serv Res. 2020;20:844. 10.1186/s12913-020-05691-8. 32907559 10.1186/s12913-020-05691-8 PMC7487713

[31] Kates N, Craven M, Bishop J, Clinton T, Kraftcheck D, LeClair K, et al. Shared mental health care in Canada. Can J Psychiatry 1997;42. 10.1177/070674379704200819. 10.1177/070674379704200819 9417365

[32] Ashcroft R, Menear M, Greenblatt A, Silveira J, Dahrouge S, Sunderji N, et al. Patient perspectives on quality of care for depression and anxiety in primary health care teams: a qualitative study. Health Expect. 2021;24:1168–77. 10.1111/hex.13242. 33949060 10.1111/hex.13242 PMC8369101

[33] Fitzpatrick SJ, Perkins D, Handley T, Brown D, Luland T, Corvan E. Coordinating mental and physical health care in rural Australia: an integrated model for primary care settings. Int J Integr Care. 2018;18:1–9. 10.5334/ijic.3943. 10.5334/ijic.3943 PMC6095085 30127703

[34] McElheran W, Eaton P, Rupcich C, Basinger M, Johnston D. Shared mental health care: the Calgary model. Fam Syst Health. 2004;22:424–38. 10.1037/1091-7527.22.4.424. 10.1037/1091-7527.22.4.424

[35] Moore JA, Karch K, Sherina V, Guiffre A, Jee S, Garfunkel LC. Practice procedures in models of primary care collaboration for children with ADHD. Fam Syst Health. 2018;36:73–86. 10.1037/fsh0000314. 29215904 10.1037/fsh0000314

[36] Sanchez K, Adorno G. “It’s like being a well-loved child”: Reflections from a collaborative care team. Prim Care Companion CNS Disord 2013;15. 10.4088/PCC.13m01541. 10.4088/PCC.13m01541 PMC3977771 24800122

[37] Lyon RK, Slawson JG. An organized approach to chronic disease care. Fam Pract Manag. 2011;18:27–31. 21842806

[38] Misra-Hebert AD, Perzynski A, Rothberg MB, Fox J, Mercer MB, Liu X, et al. Implementing team-based primary care models: A mixed-methods comparative case study in a large, integrated health care system. J Gen Intern Med. 2018;33:1928–36. 10.1007/s11606-018-4611-7. 30084018 10.1007/s11606-018-4611-7 PMC6206362

[39] Scanlon DP, Hollenbeak CS, Beich J, Dyer A-M, Gabbay RA, Milstein A. Financial and clinical impact of team-based treatment for Medicaid enrollees with diabetes in a federally qualified health center. Diabetes Care. 2008;31:2160–5. 10.2337/dc08-0587. 18678609 10.2337/dc08-0587 PMC2571067

[40] Emerson MR, Huber M, Mathews TL, Kupzyk K, Walsh M, Walker J. Improving integrated mental health care through an advanced practice registered nurse-led program: challenges and successes. Public Health Rep. 2023;138:22S–28S. 10.1177/00333549221143094. 37226950 10.1177/00333549221143094 PMC10226067

[41] Kates N, McPherson-Doe C, George L. Integrating mental health services within primary care settings: the Hamilton family health team. J Ambulatory Care Manage. 2011;34:174–82. 10.1097/JAC.0b013e31820f6435. 21415615 10.1097/JAC.0b013e31820f6435

[42] Paquette-Warren J, Vingilis E, Greenslade J, Newnam S. What do practitioners think? A qualitative study of a shared care mental health and nutrition primary care program. Int J Integr Care. 2006;6. 10.5334/ijic.164. 10.5334/ijic.164 PMC1602056 17041680

[43] Roblin DW, Howard DH, Junling R, Becker ER. An evaluation of the influence of primary care team functioning on the health of Medicare beneficiaries. Med Care Res Rev. 2011;68:177–201. 10.1177/1077558710374619. 20829237 10.1177/1077558710374619

[44] Wranik WD, Price S, Haydt SM, Edwards J, Hatfield K, Weir J, et al. Implications of interprofessional primary care team characteristics for health services and patient health outcomes: A systematic review with narrative synthesis. Health Policy. 2019;123:550–63. 10.1016/j.healthpol.2019.03.015. 30955711 10.1016/j.healthpol.2019.03.015

[45] Vader K, Donnelly C, Lane T, Newman G, Tripp DA, Miller J. Delivering team-based primary care for the management of chronic low back pain: An interpretive description qualitative study of healthcare provider perspectives. Can J Pain. 2023;7:2226719. 10.1080/24740527.2023.2226719. 37701549 10.1080/24740527.2023.2226719 PMC10494733

[46] Goodwin R, Hendrick P. Physiotherapy as a first point of contact in general practice: A solution to a growing problem? Prim Health Care Res Dev. 2016;17:489–502. 10.1017/S1463423616000189. 27263326 10.1017/S1463423616000189

[47] Howatson A, Clare W, Turner-Benny P. The contribution of dietitians to the primary health care workforce. J Prim Health Care. 2015;7:324–32. 10.1071/HC15324. 26668838 10.1071/HC15324

[48] Richardson J, Letts L, Chan D, Stratford P, Hand C, Price D, et al. Rehabilitation in a primary care setting for persons with chronic illness – a randomized controlled trial. Prim Health Care Res Dev. 2010;11:382–95. 10.1017/S1463423610000113. 10.1017/S1463423610000113

[49] McColl M-A, Shortt S, Godwin M, Smith K, Rowe K, O’Brien P, et al. Models for integrating rehabilitation and primary care: A scoping study. Arch Phys Med Rehabil. 2009;90:1523–31. 10.1016/j.apmr.2009.03.017. 19735780 10.1016/j.apmr.2009.03.017

[50] Carryer J, Yarwood J. The nurse practitioner role: Solution or servant in improving primary health care service delivery. Collegian. 2015;22:169–74. 10.1016/j.colegn.2015.02.004. 26281404 10.1016/j.colegn.2015.02.004

[51] Chouinard V, Contandriopoulous D, Perroux M, Larouche C. Supporting nurse practitioners’ practice in primary healthcare settings: A three-level qualitative model. BMC Health Serv Res 2017;17. 10.1186/s12913-017-2363-4. 10.1186/s12913-017-2363-4 PMC5485609 28651529

[52] Perry C, Thurston M, Killey M, Miller J. The nurse practitioner in primary care: alleviating problems of access?. Br J Nurs. 2013;14:255–9. 10.12968/bjon.2005.14.5.17659. 10.12968/bjon.2005.14.5.17659 15902037

[53] Sharma AE, Grumbach K. Engaging patients in primary care practice transformation: Theory, evidence and practice. Fam Pract. 2017;34:262–7. 10.1093/fampra/cmw128. 28034916 10.1093/fampra/cmw128 PMC6080566

[54] Egede L. Disease-focused or integrated treatment: diabetes and depression. Med Clin North Am. 2006;90:627–46. 10.1016/J.MCNA.2006.04.001. 16843766 10.1016/J.MCNA.2006.04.001

[55] Oni T, McGrath N, BeLue R, Roderick P, Colagiuri S, May CR, et al. Chronic diseases and multi-morbidity–a conceptual modification to the WHO ICCC model for countries in health transition. BMC Public Health. 2014;14:575. 10.1186/1471-2458-14-575. 24912531 10.1186/1471-2458-14-575 PMC4071801

[56] Coleman K, Wagner E, Schaefer J, Reid R, LeRoy L. Redefining primary care for the 21st century. White paper. vol. 16. Rockville (MD): AHRQ Publication; 2016.

[57] Jackson GL, Powers BJ, Chatterjee R, Bettger Prvu J, Kemper RA, Hasselblad V, et al. The Patient-Centered Medical Home: A systematic review. Ann Intern Med. 2013;158:169–78. 10.7326/0003-4819-158-3-201302050-00579. 24779044 10.7326/0003-4819-158-3-201302050-00579

[58] Peek CJ, National Integration Academy Council. Lexicon for behavioral health and primary care integration: concepts and definitions developed by expert consensus. Agency for Healthcare Research and Quality. 2013.

[59] Chauhan M, Niazi SK. Caring for patients with chronic physical and mental health conditions: Lessons from TEAMcare and COMPASS. Focus. 2017;15:279–83. 10.1176/appi.focus.20170008. 31975858 10.1176/appi.focus.20170008 PMC6519546

[60] Doherty WJ, McDaniel SH, Baird MA. Five levels of primary care/behavioral healthcare collaboration. Behav Healthc Tomorrow. 1996;5:25–7. 10161572

[61] Wagner EH, Austin BT, Von Korff M. Organizing care for patients with chronic illness. Milbank Q. 1996;74:511–44. 8941260 10.2307/3350391

